# Characterization of the Mouse Neuroinvasiveness of Selected European Strains of West Nile Virus

**DOI:** 10.1371/journal.pone.0074575

**Published:** 2013-09-18

**Authors:** Stephanie M. Lim, Penelope Koraka, Sander van Boheemen, Jouke M. Roose, Dick Jaarsma, David A. M. C. van de Vijver, Albert D. M. E. Osterhaus, Byron E. E. Martina

**Affiliations:** 1 Department of Viroscience, Erasmus Medical Center, Rotterdam, the Netherlands; 2 Department of Neuroscience, Erasmus Medical Center, Rotterdam, the Netherlands; Washington University, United States of America

## Abstract

West Nile virus (WNV) has caused outbreaks and sporadic infections in Central, Eastern and Mediterranean Europe for over 45 years. Most strains responsible for the European and Mediterranean basin outbreaks are classified as lineage 1. In recent years, WNV strains belonging to lineage 1 and 2 have been causing outbreaks of neuroinvasive disease in humans in countries such as Italy, Hungary and Greece, while mass mortality among birds was not reported. This study characterizes three European strains of WNV isolated in Italy (FIN and Ita09) and Hungary (578/10) in terms of *in vitro* replication kinetics on neuroblastoma cells, LD_50_ values in C57BL/6 mice, median day mortality, cumulative mortality, concentration of virus in the brain and spinal cord, and the response to infection in the brain. Overall, the results indicate that strains circulating in Europe belonging to both lineage 1 and 2 are highly virulent and that Ita09 and 578/10 are more neurovirulent compared to the FIN strain.

## Introduction

West Nile virus (WNV) is a positive-sense single-stranded RNA virus, which belongs to the genus Flavivirus. WNV is transmitted by infected mosquitoes and is maintained in an enzootic cycle between mosquitoes and birds, but can also infect and cause disease in horses and humans, which serve as incidental dead-end hosts. Previously, WNV was considered an Old World virus being endemic in parts of Africa, Europe, the Middle East, and Asia [1]. However, in 1999, WNV emerged in New York City in the United States and has since rapidly spread across North America, Mexico, South America, and the Caribbean [2-4]. No vaccines or specific therapy are currently registered for use in humans.

WNV has caused sporadic outbreaks in Central, Eastern and Mediterranean Europe for over 45 years. Phylogenetically WNV strains are classified into two major lineages. Lineage 1 constitutes strains from North America, Africa, the Middle East, Asia, Australia (Kunjin virus) and Europe. Lineage 2 strains were restricted to sub-Saharan Africa. This genetic classification has been used frequently to classify WNV obtained during outbreaks. In this respect, many strains isolated from patients with neuroinvasive disease have been classified as lineage 1. In 2008, an outbreak affected small numbers of wild birds, horses and humans in eight provinces in three regions of Italy [5,6]. Subsequently, in 2009, a new epidemic was reported in the same region, as well as in other neighboring regions in Italy, with up to 17 confirmed cases of WNV neuroinvasive disease [7]. Several strains of WNV were isolated from human specimens and sequenced. Phylogenetic analyses on the basis of the E and NS3/NS5 revealed that these strains belong to lineage 1, clade 1a, and constitute a distinct group within the western Mediterranean cluster [8]. In 2004, a lineage 2 strain was isolated from birds of prey in Hungary [9], which established itself in the region and largely spread throughout the country and into eastern Austria by 2008 [10]. During this outbreak, cases of human neuroinvasive disease were comparatively rare and rather mild with no deaths reported [10]. Recently, Greece became the focus of a large outbreak in summer-autumn 2010 [11]. Up to October 4th, 2010, 192 cases of neuroinvasive disease in humans, including 32 deaths, had been laboratory diagnosed, all in the elderly. *Culex* *pipiens* mosquitoes trapped in Nea Santa were found to be positive for WNV RNA, and sequencing of the NS5 gene gave the first indication that this virus belongs to lineage 2, and that it is highly similar to the strain that emerged in Hungary in 2004 [10]. As of November 2012, 237 confirmed human cases have been reported in the European Union (EU), of which 161 cases were in Greece, 50 in Italy, 14 in Romania and 12 in Hungary (http://www.ecdc.europa.eu/en/healthtopics/west_nile_fever/West-Nile-fever-maps/Pages/index.aspx). These outbreaks were caused by both lineage 1 and 2 strains of WNV.

It is remarkable that many of the outbreaks in humans caused by lineage 1 and 2 were not preceded by massive bird mortality. WNV-induced wild bird mortality has been described in Europe but much less intensively compared to the US. The outbreak in 1998 in Israel and 1999 in New York were the first ones where mortality among birds was reported. Because of the higher incidence of WNV neuroinvasive disease seen during the US outbreak, it was hypothesized that the introduced strain was more virulent. The complete genomic sequencing of the bird and human virulent IS-98 and NY99 strains of WNV revealed that both isolates belong to the same phylogenetic clade, sharing more than 99.8% nucleotide similarity [12]. We wished to characterize and determine the virulence profile of the European-derived WNV strains. Virulence for WNV has often been associated with envelope (E) protein glycosylation [13] and glycosylation of the NS1 protein [14]. Other virulence factors described for WNV include tropism, induction of rapid cell death, resistance to interferon, quasispecies generation and up-regulation of MHC class I expression [15]. It is therefore clear that virulence is a multi-factorial process and that many aspects need to be studied in order to elucidate the pathogenic force of viruses. Several parameters can be used to describe virulence. *In vivo* surrogate markers of virulence include immune-interfering properties, lethal dose 50 (LD_50_), median survival time (ST_50_), tropism for particular cells or tissues, as well as the viral burden present in infected tissues. In the present study we characterized the virulence of three selected European strains of WNV *in vitro* and also *in vivo* by infection of C57BL/6 mice with different doses of these virus strains. We compared their LD_50_, ST_50_, cell tropism and pathology in the brain, as well as the response to infection in the brain.

## Materials and Methods

### Ethics statement

All animal experiments described in this paper have been conducted according to Dutch guidelines for animal experimentation and approved by the Animal Welfare Committee of the Erasmus Medical Center, Rotterdam, The Netherlands. All efforts were made to minimize animal suffering. The Dutch Animal Experimentation Act (1977) demands that research establishments request a license from the Ministry of Welfare, Public Health and Cultural Affairs before carrying out any experiment. Research plans must be approved by local ethical review committees that consider the benefit of an experiment and whether this justifies the distress caused to the animals used in the procedure. The pain assessment is prospective and a system of research plan review based on the cost-benefit principle is also in place. A statistical reporting system of all animal experimentation provides the opportunity to count the number of experiments involving pain or distress to the animals with or without pain relief drugs.

### Cells and viruses

Vero E6 cells were cultured in DMEM with 10% heat-inactivated fetal bovine serum (HI-FBS), supplemented with 0.75% sodium bicarbonate and 10 mM hepes buffer. C6/36 insect cells were cultured in Leibovitz-15 medium supplemented with 5% HI-FBS, 10% tryptose phosphate broth, 0.75% sodium bicarbonate and 10 mM hepes buffer. All media were supplemented with antibiotics (100 U penicillin, 100 µg/ml streptomycin) and 2 mM L-glutamine. Cell culture reagents were obtained from LONZA (Lonza Benelux BV, Breda, the Netherlands). All cell lines tested negative for mycoplasma using a PCR assay as described [16]. Viruses used in this study and passage history were as follows: two lineage 1 Italian strains, FIN (a kind gift from Dr. Vittorio Sambri, University of Bologna, Italy; P2 on Vero E6) and Ita09 (accession GU011992.2, kindly provided by Dr. Louisa Barzon, University of Padova, Italy; P1 on Vero E6) and the Hungarian lineage 2 strain 578/10 (accession KC496015, a kind gift from Dr. Tamás Bakonyi, Szent István University, Hungary; P2 on Vero E6) isolated from the brain of a horse that died of WNV-neuroinvasive disease. Virus stocks used for this study were prepared by growing the viruses once on C6/36 insect cells and viral titers were determined on Vero E6 cells using the Spearman & Kärber method [17,18] after determining cytopathic effects five days post inoculation.

### Sequencing of the envelope gene of WNV strains

Viral RNA was isolated from C6/36 derived viral stocks using the MagnaPure LC robot system and the Total Nucleic acid isolation kit according to the manufacturer’s instructions (Roche, Almere, The Netherlands). Primers specific for the E protein were designed using the Primer Select module of DNASTAR software (DNASTAR, Madison WI, USA) and adjusted manually to obtain highest similarity with NY99 (Table S1). cDNA was synthesized using specific primers and Superscript III RT enzyme (Invitrogen, Breda, The Netherlands) and subsequently PCR-amplified using Taq DNA polymerase (Invitrogen) according to the instructions of the manufacturer. DNA fragments were gel-purified and cloned into the pCR4-TOPO vector (Invitrogen). The cloning reaction products were transformed into *E. coli* (One-Shot Top 10 competent cells; Invitrogen). Positive transformed bacteria were identified by PCR using M13 primers and sequenced using specific primers (Table S1). Five bacterial clones were selected to determine the consensus sequence of the virus stocks. Sequencing was performed in an ABI3130XL sequencer using ABI PRISM Big Dye® Terminator (Applied Biosystems, Bleiswijk, The Netherlands). Sequences were analyzed using the SeqMan module of DNASTAR software and aligned to a reference strain (original sequence of isolate deposited in GenBank) so that the E protein of the different strains was obtained from the consensus sequence of five bacterial colonies. The GenBank sequence for the original FIN isolate had not yet been deposited; therefore a closely related Italian (JF719067) sequence that gave at least 99% identity in BLAST was used as a reference instead.

### Sequencing the complete genome of WNV-FIN

RNA was isolated from the WNV-FIN strain (P2 on Vero E6) with the High Pure RNA isolation kit (Roche) according to the instructions of the manufacturer. cDNA was synthesized using random hexamer primers (Invitrogen) or a reverse primer spanning the last 24 nucleotides of the 3’ UTR of published WNV sequences, as well as Superscript III RT enzyme (Invitrogen). Fifteen sets of primers spanning the complete genome sequence of WNV were designed in conserved areas. Primers were designed using the PrimerSelect module of DNASTAR software (DNASTAR, Madison WI, USA). Primer sequences are available from the authors upon request. cDNA was amplified using PfuUltra II Fusion HS DNA Polymerase (Stratagene) and DNA fragments were purified from gel and sequenced directly in an ABI3130XL sequencer using the same primers as used for PCR amplification. Sequences were analysed using the SeqMan module of DNASTAR software.

### Next generation sequencing (NGS)

Primers were designed for the E gene that allowed five fragments of sizes between 200-400 nucleotides with about 50 nucleotides of overlap to be generated (Table S1). RT-PCR was conducted using random primers (Invitrogen) and Superscript III (Invitrogen), and DNA amplification was performed using the specific primers and PFU polymerase (Invitrogen). Fragments were gel-purified using QIAquick gel extraction kit (Qiagen, Venlo, The Netherlands) and were organized in libraries of equal concentration. Libraries were created for each virus without DNA fragmentation (GS FLX Titanium Rapid Library Preparation, Roche), emPCR (Amplification Method Lib-L) and GS junior sequencing runs were performed according to instructions of the manufacturer (Roche). Amplicons were sequenced in a blinded fashion using 454 technology. Reads from the GS-FLX sequencing data were sorted by bar code and aligned to reference sequences using CLC Genomics software 4.6.1. Using the alignment, a SNP table was made with a minimum coverage of 10 reads and a minimum variant frequency of 1.0%. Raw nucleotide sequences were filtered, aligned, trimmed and translated using pre-specified criteria applied uniformly so that all the protein E sequences used in the analyses spanned the exo-domain and the transmembrane region.

### Replication kinetics of WNV-FIN, Ita09 and 578/10 viruses

The replication kinetics of WNV-FIN, Ita09 and 578/10 were studied *in vitro* by means of a one-step growth experiment using a multiplicity of infection (MOI) of 5. N2a cells were cultured overnight in 96-well flat bottom culture plates (10^5^ cells/well) and virus was added. Viruses were allowed to adsorb for one hour at 37 °C. Cells were subsequently washed three times with serum-free medium to remove virus inoculums, replenished with fresh medium and cultured at 37 °C for 24 hours. Culture supernatants were collected in triplicate at time points 0 and 6 followed by sampling every 2 hours up to 24 hours, and were subsequently stored in -80 °C until virus titer determination. Studies of replication kinetics were conducted in parallel to eliminate any confounding effects of host cell culture.

Several parameters were determined using the results of the one-step growth experiment. The approximate eclipse period was defined as the time point before infectious virus was detected in the supernatant. The latent period (LT_50_) was defined as the time point at which half the number of virus progeny has been released into the environment and was determined by use of curve fitting to the data points by least squares (ordinary) fit. Replication rate (RR) is the slope obtained by the linear regression of the natural logarithm (ln) of the titer against time during the period of exponential growth.

### Mouse infection and survival studies

Six-week old (age-matched) female C57BL/6 mice (Harlan Laboratories B.V., Venray, The Netherlands) were inoculated intraperitoneally (i.p.) with several doses of each three virus strains (n=8 for each dose). Mice were euthanized by cervical dislocation under isoflurane anaesthesia when they reached humane end-points (immobility and paralysis), after which the brain was immediately collected for further processing. At 14 days after infection, the end-point for the survival experiment was reached and the survival rate was analyzed, and LD_50_ was calculated according to the Reed & Muench [19] and the Probit method [20]. Mice were maintained in specific pathogen-free conditions, had a 12-hour day-night cycle and were fed *ad libitum*. Serology studies were conducted using enzyme-linked immunosorbent assay (ELISA).

### Quantitation of virus in the brain

RNA copy numbers were quantified using a standard curve of *in vitro* transcribed RNA of known quantities. Run-off transcripts were generated from a plasmid containing the sequence of the 3’ UTR of WNV-NY99. Plasmid was linearized and run-off transcripts were generated using the Ambion® MaxiScript T7/T3 kit (Invitrogen). The product was digested with DNase to remove residual DNA and the reaction was cleaned up using the Qiagen RNeasy Minikit (Qiagen). *In vitro* transcribed RNA was diluted to a concentration at which DNA was no longer detected. In order to quantify viral burden in the brain, half the brain was weighed and homogenized using a metal bead in 1 mL of DMEM containing antibiotics (100 U penicillin, 100 µg/mL streptomycin). RNA copy numbers in the brain homogenates were determined using qRT-PCR with the Taqman® EZ RT-PCR kit (Applied Biosystems) and primers and probe located on the 3’ UTR of WNV (Table S1). Infectious titers in the brain were determined by titration of the brain homogenates on Vero E6 cells and calculation of the TCID_50_.

### Immunohistology

Sagittal brain and transverse spinal cord 4-µm thick paraffin sections were processed for streptavidin-biotin-peroxidase immunohistochemistry and immunofluorescence of virus nonstructural protein and cell-type markers. Sections were deparaffinized in xylene, rehydrated in descending concentrations of ethanol and incubated for 10 min in 3% H_2_O_2_ diluted in PBS to block endogenous peroxidase activity. Antigen exposure was performed by incubation for 15 min at 121 °C in citrate buffer (0.01 M, pH 6.0). Sections were incubated overnight at 4 °C with one of the following primary antibodies: goat anti-WNV NS3 (1:100; R&D Systems, Abingdon, UK), rabbit anti-human CD3 (T cell marker; 1:100; Dako, Eindhoven, Netherlands), rabbit anti-GFAP (astrocyte marker, 1:500; ZYMED, Breda, The Netherlands), and rabbit anti-Iba1 (microglial marker, 1:500; WAKO, Ochten, The Netherlands). For immunohistochemistry, primary antibodies were detected with secondary goat anti-rabbit IgG-PO, rabbit anti-goat IgG-PO (Dako) or biotinylated goat anti-polyvalent/streptavidin peroxidase (Thermo Scientific, Etten-Leur, The Netherlands) antibodies. Sections were counterstained with Mayer’s hematoxylin and mounted with Kaiser’s glycerin-gelatin and analyzed using a light microscope.

For double staining, immunofluorescence sections were incubated with goat anti-WNV NS3, and either rabbit anti-Iba1, rabbit anti-GFAP or rabbit anti-NeuN (neuronal marker, 1:500; Millipore, Amsterdam, The Netherlands) was used. Secondary antibodies directly conjugated to Alexa Fluor 488 (donkey anti-goat) and 555 (donkey anti-rabbit) (Invitrogen) were used. Nuclei were stained with DAPI. Fluorescence staining was analyzed using a Zeiss LSM 700 confocal microscope.

### Determination of inflammatory and cell death markers by qRT-PCR

RNA was isolated from brain homogenates of infected mice using the MagnaPure LC system according to the manufacturer’s instructions. cDNA was synthesized using oligo dT primer (Invitrogen) and Superscript III enzyme (Invitrogen) according to the instructions of the manufacturer. Primers specific for matrix metalloprotease (MMP)-3, MMP-9, tumour necrosis factor (TNF)-α, neuronal pentraxin (Nptx)-1,-2 and pentraxin-related protein (Ptx)-3 (Applied Biosystems) were used in PCR amplification, and mRNA copy numbers were quantified relative to β-actin using the following formula: (2^-quantity^) *100000. Quantity was determined by subtracting the Ct value of β-actin from the Ct value of the specific marker.

### Statistical analysis

P-values equal to or less than 0.05 were considered to be statistically significant. Survival curves were analyzed with the Log-rank Test, differences between viral loads and differences in expression of inflammatory and apoptotic markers were assessed using the Mann-Whitney *U* test.

## Results

### The E protein of different WNV strains is not significantly affected by *in vitro* culture

To determine whether generation of virus stocks, through one extra passage on insect cells, resulted in changes in the consensus sequence of the respective viruses, the E protein of the different virus stocks was sequenced using the Sanger method and sequences were compared to those deposited in GenBank. All virus stocks were 99% identical to the sequences of the low passage isolates deposited in GenBank (Figure S1). There were no amino acid changes found in Ita09 and 578/10 compared to the sequences deposited in Genbank. The FIN isolate, was compared to the highly similar sequence in Genbank because FIN was not sequenced before. A conservative amino acid substitution (histidine to tyrosine) was found at position 371. The lineage 2 strain from Hungary (578/10) differed by 20 amino acid substitutions from the lineage 1 strains. We have determined the complete sequence of the FIN isolate used in this study and the sequence was deposited in GenBank (accession: KF234080).

### Differences in amino acid sequence of closely related strains WNV-FIN and Ita09

As the lineage 1 strains FIN and Ita09 are very closely related and completely identical on a nucleotide level in terms of the envelope, the complete genome of FIN was compared with Ita09. The complete genome of the WNV-FIN strain (KF234080) was 99.7% identical to the nucleotide sequence of Ita09 (GU011992.2). Specifically, nine conservative nucleotide substitutions were observed throughout the genome compared to Ita09. In addition, three non-conservative amino acid differences were observed in NS3, including the proline (Ita09) to threonine (FIN) at position 249, a threonine (Ita09) to isoleucine (FIN) change at position 267 and a histidine (Ita09) to glutamine (FIN) change at position 488.

### Dominant virus variants were recovered from viral stocks and the brain of infected mice

Measuring the virulence of individual variants within a virus stock may misrepresent the virulence of a quasispecies. Since the virus population diversity is an important component of virulence, we next characterized the population diversity in our stocks using NGS (deep sequencing). Between 30-47 × 10^3^ reads were obtained per sample from the virus stocks. Given the sequence heterogeneity within protein E, the use of strain-specific primer sets with degeneracy or located in conserved regions resulted in efficient amplification. The reads generated during sequencing were aligned using the reference sequences deposited in GenBank. The viral RNA sequences recovered from the brains yielded an average of 23-38 × 10^3^ reads per sample. Coverage of the amplicons was heterogenous and ranged from 2,950 to 30,675 reads for RNA sequences recovered from the viral stocks, and 1,079 to 28,362 reads for viral RNA sequences recovered from the brain. After the filtering steps, >99.7% of the original sequences were retained.

**Figure 1:**
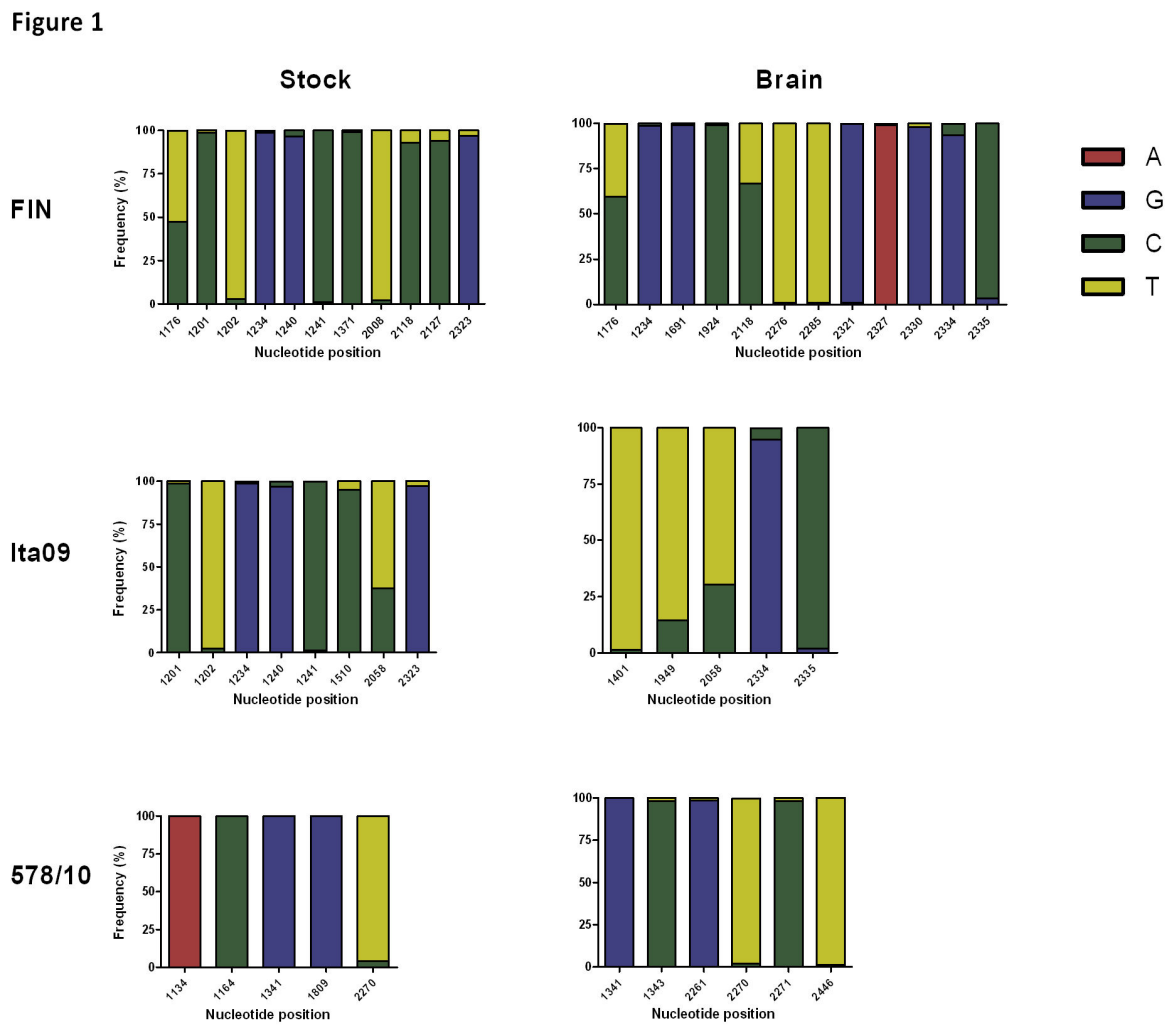
Virus variants recovered from the different WNV stocks and from the brains of infected mice. Sequences of glycoprotein E were obtained using Next Generation Sequencing (NGS) and aligned with reference sequences deposited in GenBank. Variant frequencies are indicated by nucleotide substitution at a particular reference position.

In FIN virus stock, 52.4% of the baseline viral population consisted of the T form and 47.6% of the C form at reference position 1176 (Figure 1), leading to a conservative amino acid substitution (H371Y). Furthermore, at position 2118, 92.9% of the variants contained the C form and 7.1% of the minor mutations were of the T form. Several other low-frequency mutations were found in the viral stock, ranging from 1% to 6%. In the brain of FIN-infected animals, all the variants were in similar ratios, while the frequency of the minor variant at position 2118 increased significantly by 26.1%. This variant, C2118T, also resulted in a conservative amino acid substitution (H685Y).

The baseline viral stock of Ita09 consisted of the 62.5% T form and 37.5% of the C form at reference position 2058 (Figure 1). Several other minor variants were found, ranging from 1.1% to 4.8%. For all the variants found in the viral stock, only the predominant variants were detected in the brain of infected animals, with the exception of the 2058 variant where both mutations were found. The viral stock of 578/10 consisted of only one variant at position 2270 with 96% of the T form and 4% of the C form (Figure 1). This variant was found at a similar ratio in the brain. The results indicate that the predominant variants in all the viral stocks were replicating in the brain of infected animals and that minor variants were not preferentially selected. The results from the NGS sequencing also confirmed the consensus sequence that we acquired using the Sanger method.

### WNV-FIN, Ita09 and 578/10 have similar replication kinetics *in vitro*

**Figure 2:**
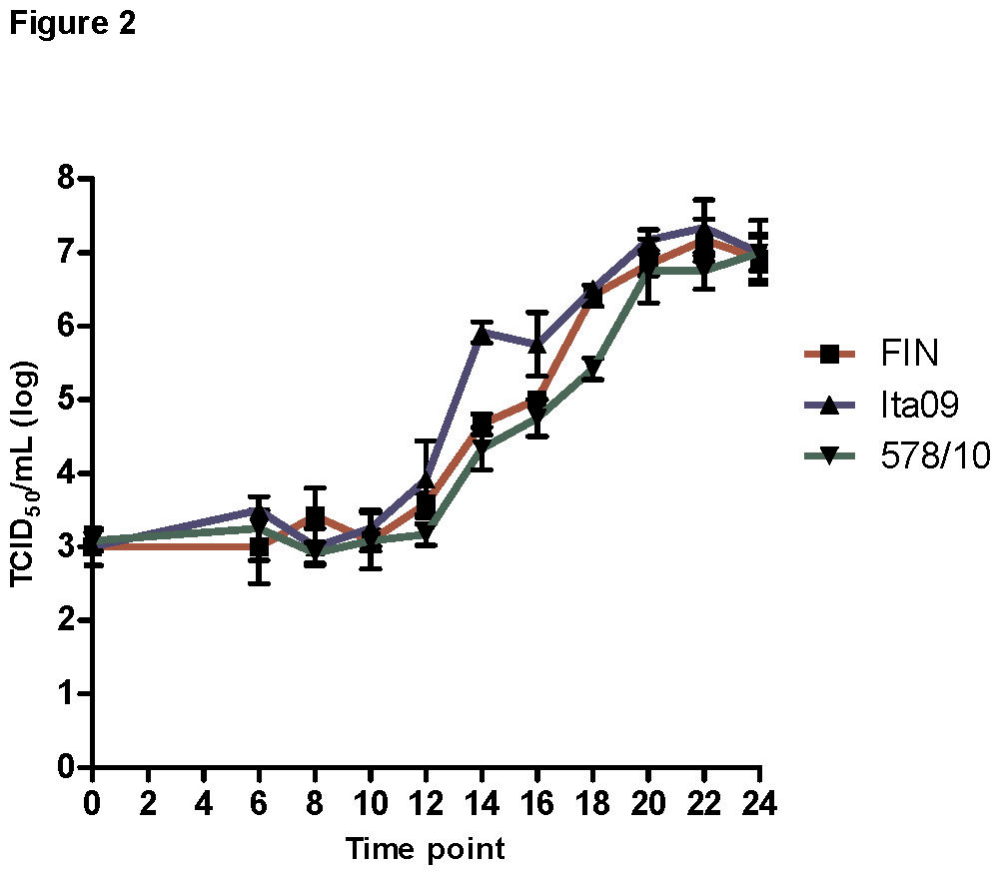
Infectious virus titers recovered from supernatant over 24 hours after infection of N2a cells with different WNV strains at different MOI. N2a cells were inoculated with WNV-FIN, Ita09 and 578/10 at an MOI of 5 TCID_50_/cell. Experiments were performed in triplicate and data represent mean ± standard deviation.

As the replicative capacity of a virus is considered a surrogate marker for virulence, we decided to compare the replication kinetics of the different WNV strains using a one-step growth experiment. This approach assumes that virulent strains produce more progeny within the host than the avirulent ones, which in turn leads to higher viral densities and consequently greater virulence levels. The one-step growth curves of the different WNV strains are summarized in Figure 2A and Table 1. Infectious virus production by the three virus strains began at approximately 14 hours post-infection. However, in order to obtain a more accurate estimate of the latent period and facilitate comparisons between the strains, we calculated the LT_50,_ which is a mathematically more robust determination of the latent period. As shown in Table 1, the RR was found to be 1.69, 1.75 and 1.71 for FIN, Ita09 and 578/10, respectively, indicating similar replication rates for the different virus strains. The LT_50_ was calculated as 16.06, 13.44 and 16.09 hours for FIN, Ita09 and 578/10, respectively. This indicates that Ita09 is released the earliest from the cell, followed by FIN and 578/10. Furthermore, the burst size of Ita09 was ten-fold higher compared to the two other strains.

**Table 1:** Analysis of the replication kinetics of WNV-FIN, Ita09 and 578/10 on N2a cells at high (5) MOI over 24 hours.

**Virus**	**Eclipseperiod (h**)	**Latent period (h**)	**LT_50_ (h**)	**Burst size (TCID_50_**)	**RR**
FIN	12	14	16,06 ± 0,02	10^1.1^	1.69 ± 0.04
Ita09	12	14	13,44 ± 0,72	10^2.1^	1.75 ± 0.05
578/10	12	14	16,09 ± 1,11	10^1.2^	1.71 ± 0.02

Experiments were carried out in triplicate and values for LT_50_ and RR are indicated as mean ± standard deviation. The LT_50_ was determined using curve fitting by least squares (ordinary) fit and the replication rate (RR) is the slope obtained by the linear regression of the natural logarithm (ln) of the titer against time during the period of exponential growth.

LT: latent time, RR: replication rate.

### Neuroinvasive properties of WNV-Ita09, FIN and 578/10 strains

**Figure 3:**
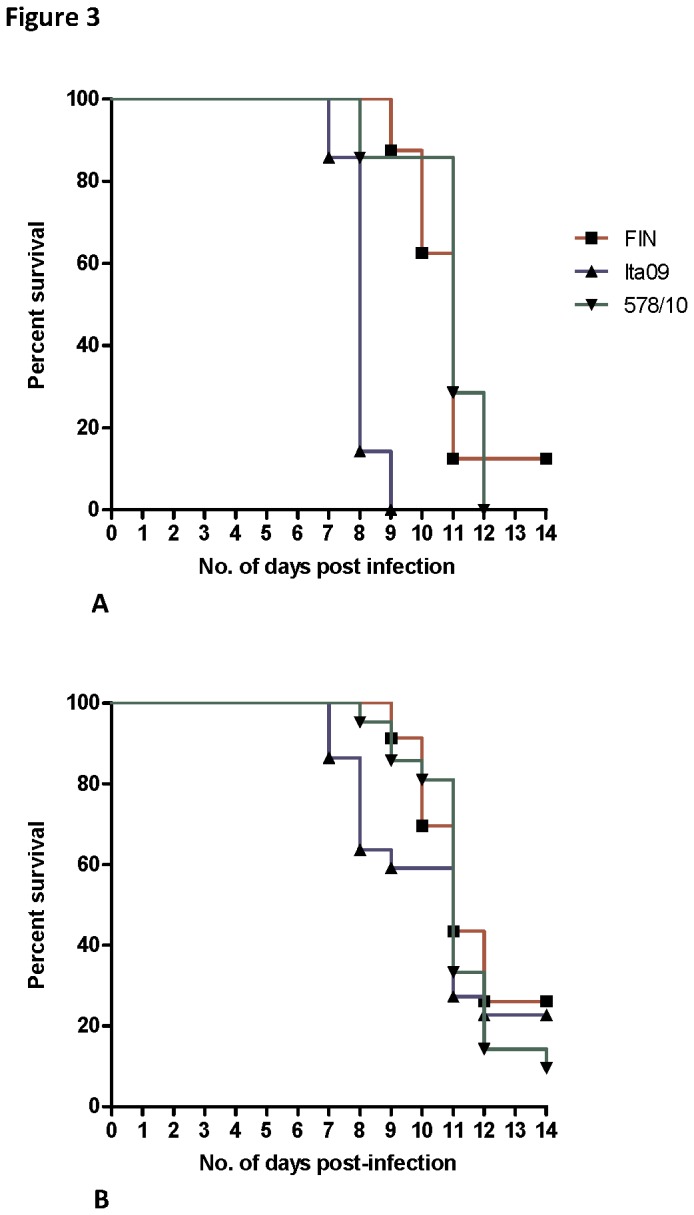
Survival curves of mice inoculated intraperitoneally with different WNV strains. (**A**) Mice were inoculated i.p. with 10^4^ TCID_50_ WNV-FIN (n=8), Ita09 (n=7) and 578/10 (n=7). Significant differences in survival were observed between FIN and Ita09 (*P* < 0.001), and Ita09 and 578/10 (*P* = 0.001). (**B**) Cumulative survival curves of mice inoculated i.p. with doses of 10^1^ TCID_50_, 10^2^ TCID_50_, and 10^4^ TCID_50_ of WNV-FIN (n=23), Ita09 (n=23) and 578/10 (n=23). No statistical significance was found between the cumulative survival curves (*P* = 0.70). Mice were euthanized between days 6-14 upon display of clinical signs of disease.

As factors such as mortality rate, *in vivo* tropism, and immune response to infection constitute important components of virulence in the dynamic host environment, we first determined the outcome of infection in 6-week old female C57BL/6 mice following i.p. infection. Mice were infected with three different doses of virus (10^4^, 10^2^ and 10^1^ TCID_50_) and differences in mortality rates were observed between the respective WNV strains (Table 2). The cumulative mortality by 14 days after challenge was higher for 578/10 (91%) than FIN (78%) and Ita09 (74%). However, comparison of the cumulative survival curves of the mice infected with all doses of the various strains revealed no statistically significant differences (Figure 3B; P = 0.70). By contrast, significantly more mortality was observed for Ita09 compared to FIN (*P* <0.001) and 578/10 (*P* = 0.001) for the 10^4^ TCID_50_ dose of virus (Figure 3A). In addition, the ST_50_ for Ita09 was shorter (8.5 days) followed by 578/10 (11 days) and FIN (11 days).

**Figure 4:**
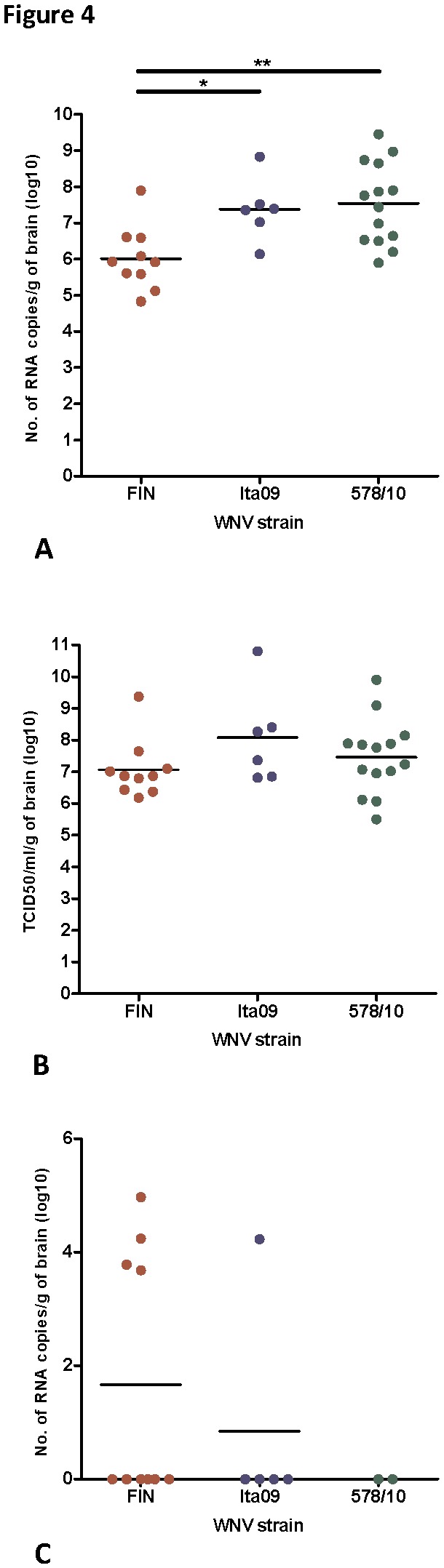
Viral burden in the brains of mice infected with WNV-FIN, Ita09 and 578/10. Mice were euthanized within 6 to 14 days upon reaching humane endpoints and viral burden was measured in terms of (**A**) RNA copies per gram of brain, and (**B**) TCID_50_ per gram of brain. (**C**) Viral RNA copies per gram of brain in mice infected with WNV-FIN, Ita09 and 578/10, and euthanized on day 20 in the absence of clinical signs of disease. * *P* <0.05, ** *P* <0.01.

**Table 2:** Mortality of 6-week old i.p. infected C57BL/6 mice using three different doses of WNV-FIN (n=23), Ita09 (n=23) and 578/10 (n=23).

**WNV strain / dose**	**First day mortality**	**Last day mortality**	**Median day mortality**	**Total mortality**	**Mortality (%**)	**Cumulative mortality (%**)
**FIN**						
10^1^ TCID_50_	9	11		4/8	50	
10^2^ TCID_50_	10	12	11	6/7	85.7	78
10^4^ TCID_50_	9	11		7/8	87.5	
**Ita09**						
10^-1^ TCID_50_^*^	12	12		1/8	12.5	
10^1^ TCID_50_	8	11		5/8	62.5	
10^2^ TCID_50_	7	12	8.5	6/8	75	74
10^4^ TCID_50_	7	9		7/7	100	
**578/10**						
10^-1^ TCID_50_^*^	8	11		3/8	37.5	
10^1^ TCID_50_	9	14		6/8	75	
10^2^ TCID_50_	9	11	11	8/8	100	91
10^4^ TCID_50_	8	12		7/7	100	

* These groups were only used to calculate the LD_50_ and were not used for determination of median day of mortality and cumulative mortality percentage.

The LD_50_ calculated using the Reed & Muench method is in agreement with the probit method, indicating similar LD_50_ values for FIN and Ita09, while considerable lower values were found for the 578/10 strain (Table 3).

**Table 3:** LD_50_ calculated with the Reed & Muench and the Probit method of WNV-FIN (n=23), Ita09 (n=23) and 578/10 (n=23) i.p. infected C57BL/6 mice.

**WNV strain**	**LD_50_ (Reed & Muench**)	**LD_50_ (Probit**)
FIN	10^0.98^ TCID_50_	10^0.75^ TCID_50_
Ita09	10^0.43^ TCID_50_	10^0.75^ TCID_50_
578/10	10^-0.42^ TCID_50_	10^-0.14^ TCID_50_

### High viral RNA load in brains of mice infected with Ita09 and 578/10 strains

We further investigated the viral burden in the brain of the mice infected with the different strains of WNV and euthanized when the humane end points were reached, within 14 days post infection. RT-PCR analysis of brain homogenates revealed high titers of viral RNA (10^5^ -10^9^) for all mice. However, mean viral RNA copies were found to be significantly higher in brains of mice infected with Ita09 and 578/10 compared to mice infected with FIN (*P* = 0.009 and *P* = 0.02, respectively) (Figure 4A). However, in terms of infectious virus titers (TCID_50_), no significant differences were observed in the brains of these mice (*P* >0.05 for all) (Figure 4B).

Mice that did not develop signs of WNV neuroinvasive disease by day 20 post infection (n=26) were considered survivors of the infection. Infection was confirmed by the fact that all these animals developed IgG antibodies to WNV (data not shown). Viral RNA was detected in the brain of nine survivors (Figure 4C). Specifically, four mice from the FIN group (one from the 10^4^, one from 10^2^ and two from the 10^1^ TCID_50_ group), and one mouse infected with 10^2^ TCID_50_ of Ita09. However, viral RNA titers were considerably lower (10^1^-10^5^) in these mice compared to those that died from infection. No significant differences were found in the number of RNA copies in the brains of these mice compared to each other (Figure 4C; P >0.05 for all). No viral RNA could be detected in the blood of any of the animals that survived WNV infection (data not shown), indicating that the detected RNA was not spillover from blood.

### Histopathology and immunohistochemistry

**Figure 5:**
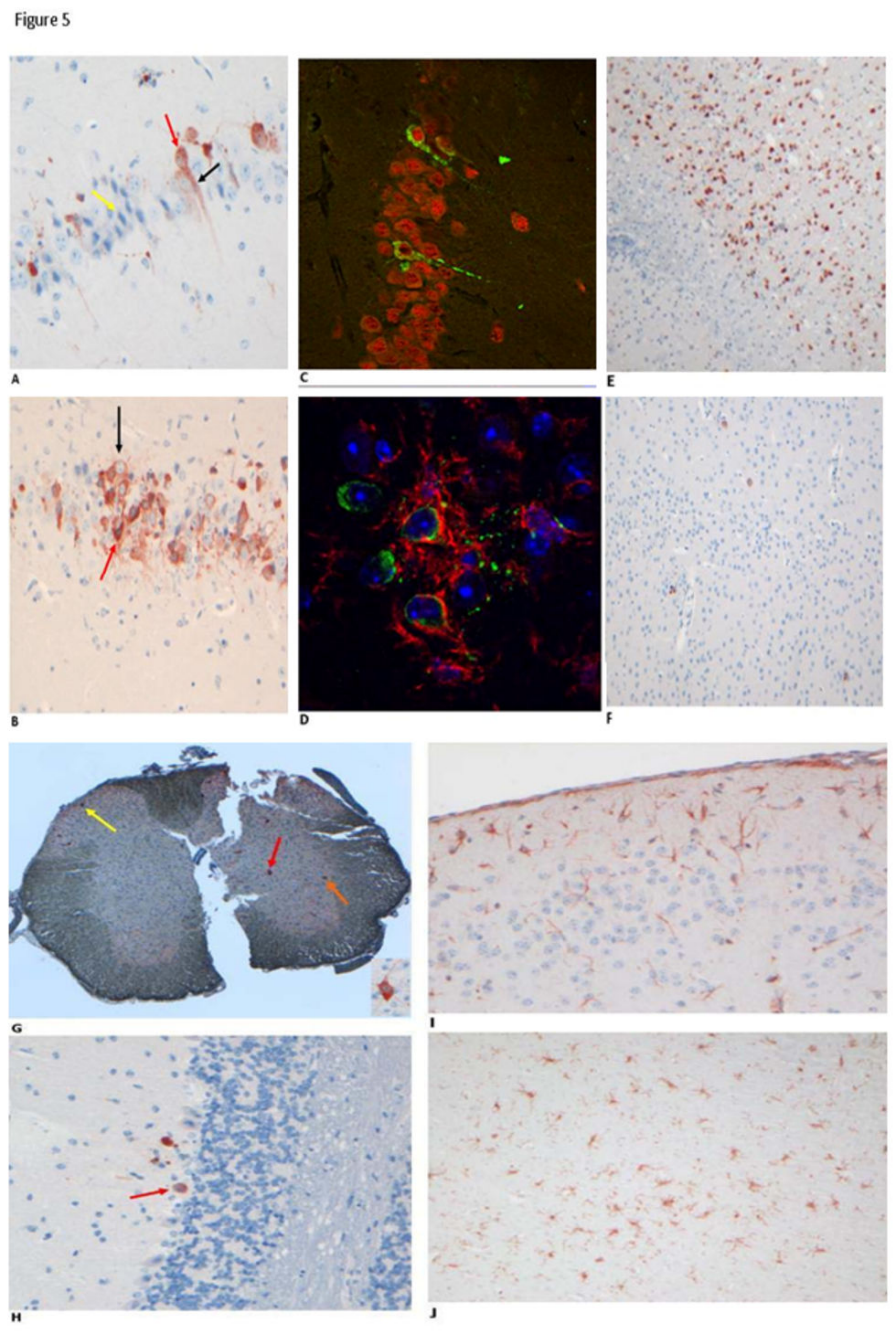
Histopathology of 6-week old C57BL/6 mice infected with WNV-FIN, Ita09 and 578/10 and euthanized upon reaching humane endpoints between days 6-14 p.i. Representative picture of (**A**) Neurons in the hippocampus of a mouse infected with Ita09, stained with anti-WNV NS3 antibody (objective 20×). Antigen is distributed in the cell body (red arrow) and proximal dendrite (black arrow). Some of the infected neurons appear to be in a healthy state with normal nuclear, perikaryal and dendritic morphologies (red/black arrow), while some uninfected neurons appear to be in a moribund state (yellow arrow). (**B**) Neurons in the hippocampus of a mouse infected with Ita09, stained with anti-WNV NS3 antibody (objective 20×). Antigen-expressing neurons appear to be in a different state of health varying from healthy appearance with normal nuclear, perikaryal and dendritic morphologies (black arrow), to dying cells (red arrow). (**C**) Double staining as seen by confocal microscopy (objective 20×) showing neurons (stained with NeuN antibody and Alexa Fluor 555 conjugate; red) infected with Ita09 (stained with anti-NS3 antibody and Alexa Fluor 488 conjugate; green). (**D**) Double staining as seen by confocal microscopy (objective 40×) showing activated microglia (stained with anti-Iba1 antibody and Alexa Fluor 555 conjugate; red) engulfing neurons infected with WNV-Ita09 (stained with anti-NS3 antibody and Alexa Fluor 488 conjugate; green). Nuclei are stained with DAPI (blue). (**E**) Neo-cortical neurons in the brain of a mouse infected with WNV-Ita09, stained with anti-WNV NS3 antibody (objective 10×). (**F**) Neo-cortical neurons in the brain of a mouse infected with WNV-FIN, stained with anti-WNV NS3 antibody (red arrows; objective 10×) (**G**) Spinal cord of a mouse infected with WNV-578/10, stained with anti-WNV NS3 (objective 4×). Infection of motor neurons (red arrow), anterior horn (orange arrow) and posterior horn (yellow arrow) was observed. Insert shows infected motor neuron (objective 20×). (**H**) Purkinje cell in cerebellum of mouse infected with Ita09 (red arrow), stained with anti-WNV NS3 antibody (objective 20×). (**I**) Mild to moderate activation of astrocytes in the cortex of a mouse infected with WNV-578/10, stained with anti-GFAP (objective 20×). (**J**) Activation of microglia cells in the cortex of the brain of a mouse infected with WNV-FIN, stained with anti-Iba1 (objective 10×).

In order to assess if the viruses differ in their tropism for particular regions of the brain, and compare the relative damage caused by the different strains, we performed immunohistochemical staining with anti-WNV-NS3 polyclonal antibody. WNV-NS3 positive cells occurred in brains and spinal cords of all mice that developed neurological signs. Most positive cells could be identified as neurons, with the antigen being distributed in the cell body and proximal dendrites (Figure 5A). Some positive cells showed features of degenerative processes such as dystrophic neurites, small cell body and pyknotic nuclei (Figure 5B). In addition, labeling was associated with unidentifiable structures likely representing neuronal debris. In order to confirm that positive cells were neurons, we performed double-labeling immunofluorescence with the neuronal marker NeuN (Figure 5C). Most positive cells were positive for NeuN, while no WNV-NS3 positive cell was positive for glial fibrillary acidic protein (GFAP, astrocytes) or Iba1 (microglia), although in many occasions WNV positive cells and debris-like structures were surrounded by Iba1 positive processes, pointing to an intimate relationship with microglia cells (Figure 5D).

**Figure 6:**
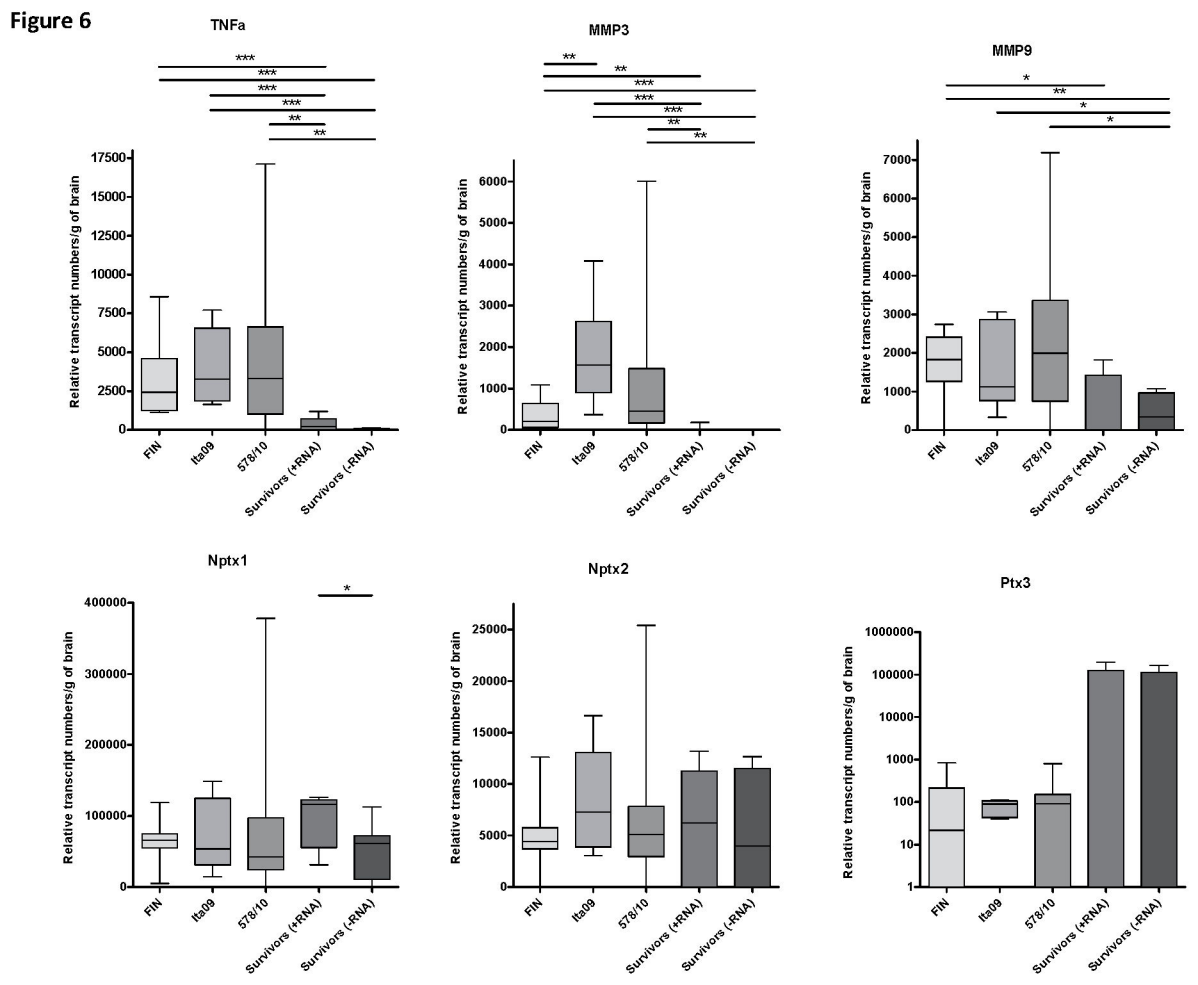
Relative number of RNA transcripts of markers in the brains of mice infected with WNV-FIN, Ita09 and 578/10. Sick animals were euthanized within days 6-14 upon reaching humane endpoints or on day 20 post-infection in the absence of clinical signs. Transcripts were compared with animals that survived WNV infection (day 20 p.i) and that were either positive or negative for viral antigen in the brain. Mean is indicated by a cross and median by a line in the boxes. The box represents the interquartile range. * *P* <0.05, ** *P* <0.01, *** *P* <0.001. Abbreviations: TNF = tumour necrosis factor; MMP = matrix metalloproteinase; Nptx = neural pentraxin; Ptx = pentraxin.

The extent of infection in the brain was determined in terms of infected brain areas (Table S2). In general, positive neurons occurred in all areas of the brain (Table S2) and the spinal cord, although the regional distribution and relative density of positive cells was highly variable between animals injected with the same virus. For instance, animals infected with Ita09 virus showed highly sporadic, moderate amounts, or very frequent NS3-positive neurons in the hippocampal CA1 area (Figure 5A and B). A similar variability was also observed in other brain areas, including the neocortex, striatum and cerebellar cortex (Figure 5E and F), and there was no correlation between the amount of positive cells in different brain areas in the same mouse. Because of the large variability between animals injected with the same viruses, it was difficult to determine whether systematic differences occurred in the amount and distribution of positive cells between animals injected with different viruses. For instance, infection of Purkinje cells in the cerebellum was observed in sections of some mice infected with Ita09 (Figure 5H) and 578/10 but not in mice infected with FIN. However, in view of the potential focal distribution of infected cells, the question whether this difference holds true for all Purkinje cells of FIN-injected mice would require the systematic analysis of the entire cerebellum of all mice, which is beyond the scope of this study. Nevertheless, based on the analyses performed in this study, mice infected with Ita09 and 578/10 showed more positive cells than mice infected with FIN.

Furthermore, antigen distribution and quantity of positive cells in the spinal cord was also highly variable, with WNV positive cells occurring in both dorsal and ventral horns. Although not systematically investigated, spinal motor neurons in the ventral horn were infected more often than dorsal horn cells, despite their relatively low abundance compared to other spinal cord cells (Figure 5G).

Analysis of WNV-NS3 expression in the brains of mice that did not develop clinical disease and that were killed at 20 days post infection revealed no positive cells, which is consistent with the absence or low abundance of virus antigen in the brains of these mice. Positive cells were also not found in the spinal cord of these animals.

To determine whether the presence of virus-positive neurons and neuronal debris correlated with microglia cell activation we stained for Iba1, which is up-regulated in activated microglia cells [21-24]. Increased Iba1 staining, as compared to control mice, was observed in the nervous system of all mice with virus-positive neurons (Figure 5J). In accord with the variability in distribution and quantity of WNV-NS3 positive neurons, changes in Iba1 staining was also variable between different mice infected with the same virus or different viruses. Remarkably, however, in some cases, areas with high levels of WNV-NS3 positive cells and debris did not show a strong increase in Iba1 positive cells, indicating that a high level of virus-infected cells is not necessarily paralleled by high levels of microglia cell activation. Astrocytosis (as demonstrated by increased GFAP-staining) was seen in all animals and cases ranged from mild to severe for all virus strains (Figure 5I). Astrocytosis and microgliosis were also evident in the brains of the survivor mice.

Finally, we analyzed infiltration of T-cells using anti-CD3 staining (Table S3). Perivascular cuffing by CD3-positive cells was highly variable and was only evident in localized regions in a subset of sections examined, consistent with the large variability of infected areas. Infiltration of the neuropil by T cells (CD3-positive cells) was mainly seen in animals infected with Ita09 in the cerebrum and brainstem (~1 positive cell per high power field [HPF]; objective 40×) and in some of the animals infected with FIN or 578/10 (<1 cell per HPF in the brainstem). Interestingly, infiltration of CD3-positive cells into the cerebrum and brainstem were found in all mice that survived infection with any of the WNV-strains. In comparison to the mice that had died from infection, all mice that survived appeared to have more CD3-positive cells in the brain. Taken together, the IHC studies provide supportive evidence that Ita09 and 578/10 are more virulent than FIN.

### Response to WNV infection in brains of mice

To allow for quantitative comparison of the response to infection with different strains of WNV, we performed qRT-PCR using half brain homogenates (Figure 6). The survivor mice were split into two groups based on presence or absence of viral RNA in the brain. We chose for markers that have been associated with viral encephalitis and neurodegenerative diseases. The inflammatory marker TNF-α was increased in the brains of mice infected with FIN (*P* = 0.002; *P* <0.0001), Ita09 (*P* = 0.007; 0.007) and 578/10 (*P* = 0.006; 0.003) compared to the survivors positive and negative for viral RNA in the brain, respectively. One of the markers involved in the breakdown of extracellular matrix, MMP-3, was significantly up-regulated in the brains of mice infected with FIN (*P* = 0.004; 0.0009), Ita09 (*P* = 0.0007; 0.0007) and 578/10 (*P* = 0.003; 0.002) compared to convalescent mice with and without viral RNA in the brain. In addition, MMP-3 transcript levels were significantly higher in Ita09 compared to those infected with FIN (*P* = 0.005). MMP-9 transcript was increased in mice infected with FIN (*P* = 0.02) and 578/10 (*P* = 0.03) only when compared to the convalescent mice positive for viral RNA in the brain. Nptx-1 (a marker involved in apoptosis) was only up-regulated in the brains of the survivor mice group with viral RNA in the brain compared to the group without viral RNA (*P* = 0.05). Nptx-2 and Ptx3, inflammatory markers involved in complement activation and complement-mediated clearance of apoptotic cells, were not significantly up-regulated in any of the experimental groups. These data identify some inflammatory markers significantly elevated during infection of the brain with WNV, but none of the examined markers correlate with virulence.

## Discussion

In this study we have characterized the European WNV strains FIN, Ita09 (both lineage 1) and 578/10 (lineage 2). Neurovirulence of the three WNV strains were determined by comparing *in vitro* replication kinetics, median day mortality, cumulative mortality, LD_50_, concentration and distribution of virus in the brain, and the response to infection in the brain.

Despite the fact that WNV has been circulating in Europe for half a century, it is only in the more recent years that this virus has caused considerable outbreaks in humans, horses and to a much lesser extent in wild birds. This is in contrast to the emergence of WNV in North America during which wild birds were heavily affected and significant numbers of human neuroinvasive disease cases with high mortality were reported. It is possible that this increase in severity is a result of the movement of the virus into areas with large immunologically naïve populations that consist of a large proportion of elderly and immunocompromised individuals [25]. However, it has also been suggested that a more virulent strain of the virus was introduced [26]. It is also hypothesized that the viruses currently circulating in Europe differ in their virulence profile compared to the North American strains.

There have been a number of explanations for why viruses are virulent [27,28], and it is clear that virulence is an adaptive process and that it is the result of the trade-offs between virus transmissibility, virus pathogenic force, and recovery potential of the host. In several models it has been shown that changes in virulence are associated with changes in different aspects of the biology of virus-host interaction, suggesting that virulence of a given virus may be affected by a potentially large number of factors (reviewed in [29-33]). Since no single general factor exists that can be used to predict the relative virulence of viruses, we investigated the virulence of European WNV strains by considering a series of parameters. Even though it is possible to investigate virulence by conducting a straight kinetic analysis and examining viral spread in the brain over time, we decided to use survival as an outcome of disease severity. This is because disease can be a direct consequence of viral burden, inflammatory response and injury and death of cells in the CNS, and therefore an appropriate measure of virulence. Following infection, WNV replicates to high levels during the acute phase, after which the virus typically enters the brain and causes meningo-encephalitis before the immune system is able to control the infection. Our aim was to define the virulence profile of European WNV strains by measuring different markers *in vitro* and *in vivo*.

First we determined the population structure (quasispecies) in our virus stocks. We chose to characterize the stock rather than the dominant variant in the stock that was used to infect mice. It has been shown that viral quasispecies is more than just a collection of mutants, but a group of interactive variants, which together contribute to pathogenesis [34]. For instance, it was found that the diversity of the quasispecies of Polio virus correlated with enhanced pathogenesis in mice [34]. Ciota et al. [35] have shown that the quasispecies in WNV populations correspond to substantial phenotypic diversity that differed in relative fitness *in vitro*. We have used NGS to determine the population phenotype of our viral stocks, based on the glycoprotein E gene. This gene was selected because it is the principal receptor that determines tropism for neurons, contains markers of virulence, and it harbors areas that allow monitoring of virus evolution. One advantage of the NGS is the possibility to detect minor variants. In this study, we found that only the dominant variants from the stock were selected in the brain. One important issue concerning the NGS is the ability to distinguish between true variants and variants detected as a result of errors introduced during PCR amplification and/or sequencing. Therefore, within our department, we have determined the error rate threshold specific for this platform. We found that a threshold of 0.026% (manuscript in preparation) is sufficient to exclude variants detected as a result of errors. The technical cut-off value of 1% described in this manuscript is therefore well above the error margin attributed to reverse transcription, amplification and sequencing errors. Furthermore, emergence of new variants was also detected in the brain, which could have been a result of mutations arising during the replication cycle. We did not specifically study whether the population structure of our strains contributed to virulence.

The infection cycle of WNV has not been studied extensively *in vitro*, so we first addressed the dynamics of WNV infection *in vitro* in neuronal cells. We found that only adherent cells could be infected and infection of N2a cells in suspension was not successful (data not shown). The reason for this phenomenon is unknown, but may be related to receptor availability on adherent cells. Currently, there is little known about the host receptor for flaviviruses. The replication cycle of WNV can be divided into three phases; (1) dispersal-diffusion-attachment phase, (2) eclipse phase (begins with infection and ends when the virus progeny matures inside the host), and (3) release phase (the virus offspring are released from the infected cell). The total number of progeny released in the supernatant is termed the burst size. Examination of these three stages in virus replication is useful, because the associated growth parameters (eclipse period, latent period, exponential growth rate, and burst) yield plausible hypotheses to account for differences in virulence.

A study has shown that large clusters of matured virus of the Sarafend strain appear at the plasma membrane, as well as in vacuoles, at 10-12 hours post-infection [36]. This is in line with our results where we determined the eclipse period to be at approx. 12 hours p.i. The study further showed that maturation of WNV at the plasma membrane, and therefore budding from infected cells, is the dominant mode of maturation for this virus, but that during the later stage of infection (from 12 hours p.i.) the virus is also released via exocytosis, most likely due to advanced cytopathic effects [36]. As a result, it may be difficult to determine the precise time point of the burst size and we have therefore additionally determined the LT_50_. Furthermore, analyzing the RR may shed light on the potential of WNV strains to overwhelm the target cells. Virions that are able to overwhelm the system quickly will have an increased chance of colonizing the remaining uninfected cells, an advantage particularly important *in vivo*. We found that Ita09 replicates faster as evidenced by the LT_50_ and release of up to ten-fold more virus after the first replication cycle.

The increase in morbidity and case-fatality rates caused by North American lineage 1 strains relative to lineage 2 strains led to the hypothesis that lineage 1 strains are highly pathogenic while lineage 2 strains that used to be endemic only to Africa are of low virulence [4]. Conversely, recent outbreaks in South Africa and Europe indicate that lineage 2 strains may also cause severe disease [37]. This observation was also supported by experimental studies in mice showing that differences in virulent WNV strains did not correlate with the phylogenetic lineage, source of isolate, geographic distribution, passage level or year of isolation, and suggest instead that pathogenicity is not genotype specific and that both lineage 1 and 2 are neurovirulent [38,39]. Our results indicate that all the European strains studied are virulent in C57BL/6 mice and that the lineage 1 strain (Ita09) and lineage 2 strain (578/10), which share similar virulence profiles, are slightly more virulent than FIN. Two lineage 1 viruses with 99.7% identity (FIN and Ita09) were found to share a different virulence profile. These viruses differed in terms of three non-conservative amino acids. One of the substitutions present in the Ita09 strain, T249P, is a mutation in the helicase domain of the NS3 protein and has been associated with increased virulence in American crows [40]. This mutation has been found in the more recent Italian WNV isolates from 2008 (15803/08 and 15217/08), while the Italy 1998-equine strain still has the threonine at this position [41]. Interestingly, the 2010 Greek isolate also contains the proline, and may be responsible for the increased virulence of this strain compared to other strains from lineage 2 [10]. However, the virulence properties of this sole substitution in outbred mice remains unclear, and its role in virulence in humans on a population level is questionable. For instance, a WNV strain isolated in 2007 from golden eagles in Spain carrying a TP mutation did not have increased pathogenicity in mice compared to other strains [42]. Similarly, the WNV-FIN strain used in this study was isolated from a patient with neuroinvasive disease. It is possible that the three amino acids differences between Ita09 and FIN collectively reduced the virulence of FIN.

Mice that had died as a result of infection with Ita09 and 578/10 were found to have a significantly higher number of RNA copies in the brain compared to those infected with FIN. Infectious virus titers, however, were not significantly different. This discrepancy might be explained by the involvement of immature virus particles, which may also play a role in the pathogenesis of WNV [43]. It is therefore possible that there was a higher viral burden in the brains of mice infected with Ita09 and 578/10 as a result of a larger amount of immature virus particles, which may explain the higher virulence observed for these virus strains.

The histopathologic findings observed in the brain and spinal cord samples of the mice that succumbed to the infection were pathognomonic, with moderate to severe infection observed in mice infected with Ita09 and 578/10 strains. In agreement with previous studies, WNV antigen was found in neurons in the spinal cord, cortex, hippocampus and brainstem [15,44]. Interestingly, antigen of Ita09 and 578/10 was found also in the Purkinje cells of the cerebellum. Infection of Purkinje cells by North American strains of WNV has been demonstrated in hamsters [45-47], birds [48,49], macaques [50] mice [51] and humans [52]. This targeting of Purkinje cells of the cerebellum has been shown to be a unique pathologic finding in WNV encephalitis, unlike the encephalitides caused by other closely related flaviviruses [48,53-57]. The higher frequency and intensity of antigen staining in the central nervous system and the higher mortality observed in the Ita09 and 578/10-infected groups compared with the FIN–infected group (lineage 1), suggest that these two strains are more virulent.

Even though some of the mice that survived infection were positive for viral RNA, IHC staining did not demonstrate antigen in the brain. The intensity of staining found using IHC did roughly correlate with the amount of RNA found in the brain. As RNA titers in the brain of the survivor mice were significantly lower compared to mice that died from infection, it is possible that these low titers, in addition to unsystematic sampling, resulted in the absence of detection of positively stained cells using IHC in brain samples of the survivor mice.

We have also measured the response to infection in the brain as a virulence factor. Although astrocytosis and microgliosis were observed in all mice that died from the infection, we did not find evidence of infection of these cells. It is believed that activated microglia and astrocytes contribute to an excessive inflammatory response, which triggers a process of secondary cell death or functional depression in structurally normal areas distant from, but connected to the original sites involved. We have found that MMP-3 transcript was significantly elevated in all animals that developed severe disease. MMPs are capable of degrading the tight junction proteins of human brain microvascular endothelial cells, thereby compromising the integrity of the blood-brain barrier. Further studies are necessary to address the question of whether MMP-3 is a virulence factor triggered by pathogenic WNV strains.

To our knowledge, this is the first study to characterize pathogenic properties of WNV strains circulating in Europe. We have found that all three European strains of WNV are neurovirulent in C57BL/6 mice; however, the data also suggest that Ita09 and 578/10 show an increased virulence in comparison to FIN. Studies are ongoing to determine the virulence of these strains in European birds and in other outbred animal models.

## Supporting Information

Figure S1 **The sequences of glycoprotein E of the WNV stock used in this study were determined with the Sanger method.** The deduced amino acid sequences were aligned. The sequence of FIN was deposited in GenBank (KC493970). The sequence of Ita09 and 578/10 were compared to GU011992.2 (Ita09) and KC496015 (578/10) and FIN was compared to a highly similar sequence (accession JF719067.1; 99% similar to FIN).
(DOCX)

Table S1 **Primers used for sequencing the envelope of WNV-NY99, FIN, Ita09 and 578/10.** Primers indicated with * were kindly provided by Dr. Tamás Bakonyi (Szent István University, Hungary) and were used to sequence the envelope of 578/10.
(DOC)

Table S2 **Antigen distribution (described in terms of staining of the NS3 protein) in the brains of mice infected with WNV-FIN, Ita09 and 578/10, and either euthanized upon display of clinical signs of disease (between days 6-14) or euthanized on day 20 without showing signs of illness.** Percentage indicates the amount of infected mice that are positive for antigen in each particular brain region.
(DOC)

Table S3 **Detection of CD3 positive cells in the brains of mice infected with WNV-FIN, Ita09 and 578/10, and either euthanized upon display of clinical signs of disease (between days 6-14) or euthanized on day 20 without showing signs of illness.** Numbers indicate the number of positive cells; HPF: high power field; objective 40X.
(DOC)
