# Supplementary figures and images for: Characterization of the Mouse Neuroinvasiveness of Selected European Strains of West Nile Virus
Source: PLoS One. 2013 Sep 18;8(9):e74575. doi: 10.1371/journal.pone.0074575 (PMC3776840; doi:10.1371/journal.pone.0074575)

**Supplementary information**

**Figure 1**


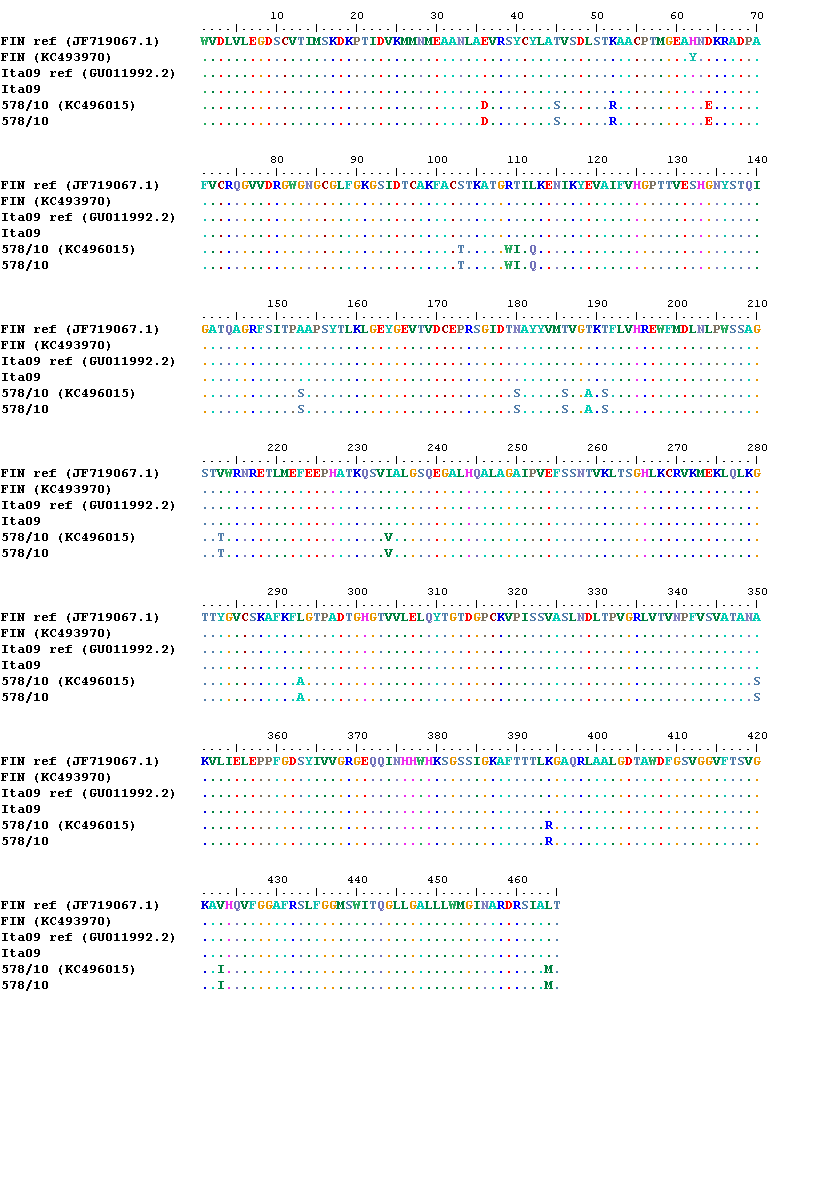

Supplement: Figure S1 — The sequences of glycoprotein E of the WNV stock used in this study were determined with the Sanger method. The deduced amino acid sequences were aligned. The sequence of FIN was deposited in GenBank (KC493970). The sequence of Ita09 and 578/10 were compared to GU011992.2 (Ita09) and KC496015 (578/10) and FIN was compared to a highly similar sequence (accession JF719067.1; 99% similar to FIN). (DOCX) [file pone.0074575.s001.docx]
