# Supplementary material for: Characterization of the Mouse Neuroinvasiveness of Selected European Strains of West Nile Virus
Source: PLoS One. 2013 Sep 18;8(9):e74575. doi: 10.1371/journal.pone.0074575 (PMC3776840; doi:10.1371/journal.pone.0074575)
Supplement: Table S1 — Primers used for sequencing the envelope of WNV-NY99, FIN, Ita09 and 578/10. Primers indicated with * were kindly provided by Dr. Tamás Bakonyi (Szent István University, Hungary) and were used to sequence the envelope of 578/10. (DOC) [file pone.0074575.s002.doc]

**Supporting Information**

**Supplementary Table 1.** Primers used for sequencing the envelope of WNV-NY99, FIN, Ita09 and 578/10. Primers indicated with * were kindly provided by Dr. Tamás Bakonyi (Szent István University, Hungary) and were used to sequence the envelope of 578/10.

| **Primer** | **Sequence (5’to 3’)** | **Application** |
| --- | --- | --- |
| #721modified48R | AGCTCTTGCCGGCTGATGTC | RT-PCR |
| #722modified51F | AGCTTCAACTGCCTTGGAATGAG | PCR / Sequencing |
| #723modified57R | TGTCAGCGTGCACGTTCACGGA | PCR / Sequencing |
| #43WNV.E04F | CGCCAAATTTGCCTGCTCTAC | Sequencing |
| #44WNV.E05R | AGTTTGAGGAACCACACGCCA | Sequencing |
| #732WNVII-870f* | CCTCGTTGCAGCTGTCATTG | Sequencing |
| #733WNVII-1630r* | TCCATGGCAGGTTCAGATCC | Sequencing |
| WNVE-Deepseq-F1 | ATGACAAACGTGCTGACC | 454 sequencing |
| WNVE-Deepseq-R2 | GTTCACAGTCCACTGTCACCTCTC | 454 sequencing |
| WNVE-Deepseq-F2 | CGCCTTCATACACACTAAAG | 454 sequencing |
| WNVE-Deepseq-R3 | AGCCTTTGAACAGACGCCAT | 454 sequencing |
| WNVE-Deepseq-F3 | GTAGAGTGAAGATGGAAAAATTGC | 454 sequencing |
| WNVE-Deepseq-R4 | GCTGTGTCTCCTAGAGCGGC | 454 sequencing |
| WNVE-Deepseq-F4 | ACAACCACCCTCAAAGGA | 454 sequencing |
| WNVE-Deepseq-F1/853-HUN | AACGAGAAAAGAGCTGACCCCG | 454 sequencing |
| WNVE-Deepseq-R2/854-HUN | GCTCACAGTCAACCGTGACCTCAC | 454 sequencing |
| WNVE-Deepseq-F4/859-HUN | CCTTTACCACTACACTCAGAGGAGCT | 454 sequencing |
| WNVE-Deepseq-723-HUN | TGTCAGCATGGACGTTGACCGA | 454 sequencing |
| 3’UTR F | CCACCGGAAGTTGAGTAGACG | Taqman |
| 3’UTR R | TTTGGTCACCCAGTCCTCCT | Taqman |
| 3’UTR probe FAM-TAMRA | TGCTGCTGCCTGCGGCTCAACCC | Taqman |
