# Supplementary material for: Characterization of the Mouse Neuroinvasiveness of Selected European Strains of West Nile Virus
Source: PLoS One. 2013 Sep 18;8(9):e74575. doi: 10.1371/journal.pone.0074575 (PMC3776840; doi:10.1371/journal.pone.0074575)
Supplement: Table S2 — Antigen distribution (described in terms of staining of the NS3 protein) in the brains of mice infected with WNV-FIN, Ita09 and 578/10, and either euthanized upon display of clinical signs of disease (between days 6-14) or euthanized on day 20 without showing signs of illness. Percentage indicates the amount of infected mice that are positive for antigen in each particular brain region. (DOC) [file pone.0074575.s003.doc]

**Supplementary Table 2**. Antigen distribution (described in terms of staining of the NS3 protein) in the brains of mice infected with WNV-FIN, Ita09 and 578/10, and either euthanized upon display of clinical signs of disease (between days 6-14) or euthanized on day 20 without showing signs of illness. Percentage indicates the amount of infected mice that are positive for antigen in each particular brain region.

| **WNV strain** | **CTX** | **CNU** | **TH** | **HY** | **HPF** | **MB** | **MY/Pons** | **CB** |
| --- | --- | --- | --- | --- | --- | --- | --- | --- |
| WNV-FIN  n=10 (died) | 60% | 20% | 10% | 40% | 30% | 30% | 20% | 40% |
| n=9 (survived) |  |  |  |  |  |  |  |  |
| WNV-Ita09  n=10 (died) | 90% | 60% | 50% | 20% | 30% | 20% | 50% | 60% |
| n=6 (survived) |  |  |  |  |  |  |  |  |
| WNV-578/10  n=18 (died) | 78% | 50% | 39% | 39% | 33% | 44% | 44% | 17% |
| n=3 (survived) |  |  |  |  |  |  |  |  |

Abbreviations: CTX = cortex; CNU = cerebral nuclei; TH = thalamus; HY = hypothalamus; HPF = hippocampal formation; MB = midbrain; MY = medulla; CB = cerebellum
