# Supplementary material for: Characterization of the Mouse Neuroinvasiveness of Selected European Strains of West Nile Virus
Source: PLoS One. 2013 Sep 18;8(9):e74575. doi: 10.1371/journal.pone.0074575 (PMC3776840; doi:10.1371/journal.pone.0074575)
Supplement: Table S3 — Detection of CD3 positive cells in the brains of mice infected with WNV-FIN, Ita09 and 578/10, and either euthanized upon display of clinical signs of disease (between days 6-14) or euthanized on day 20 without showing signs of illness. Numbers indicate the number of positive cells; HPF: high power field; objective 40X. (DOC) [file pone.0074575.s004.doc]

**Supplementary Table 3.** Detection of CD3 positive cells in the brains of mice infected with WNV-FIN, Ita09 and 578/10, and either euthanized upon display of clinical signs of disease (between days 6-14) or euthanized on day 20 without showing signs of illness. Numbers indicate the number of positive cells; HPF: high power field; objective 40X.

| **T cell staining (anti-CD3 antibody)** | | | | | | |
| --- | --- | --- | --- | --- | --- | --- |
|  | Cerebrum | | Brainstem | | Cerebellum | |
|  | Infiltration | Perivascular cuffs | Infiltration | Perivascular cuffs | Infiltration | Perivascular cuffs |
| **FIN** | <1/ HPF (in 50% of mice) | Neg | <1/ HPF | Neg | Neg | Neg |
| **Survived** |  | 1 layer thick in 10% of mice | 1 cell per HPF (10% of mice) |  |  |  |
| **Ita09** | 1/ HPF (in 50% of mice) |  | 1/HPF |  |  | <1/mouse (1 cell layer thick) |
| **Survived** | Clusters of positive cells (in 33% of mice) |  | Clusters of positive cells (in 33% of mice) | 2 cell layers thick |  |  |
| **578/10** | Neg | Neg | <1/HPF (in 50% of mice) | Neg | Neg | Neg |
| **Survived** | <1 cell per HPF (50% of the mice) |  | <1 cell per HPF (50% of the mice) |  |  |  |
